# TCR V *α*- and V *ß*-Gene Segment Use in T-Cell Subcultures Derived from a Type-III Bare Lymphocyte Syndrome Patient Deficient in MHC Class-II Expression

**DOI:** 10.1155/1992/29814

**Published:** 1992

**Authors:** M. Lambert, M. van Eggermond, F. Mascart, E. Dupont, P. van den Elsen

**Affiliations:** 1 Department of Immunohematology and Blood Bank University Hospital Leiden 2300 RC The Netherlands; 2 Department of Immunohematology Hospital St. Pierre Brussels Belgium; 3 Department of Immunohematology Erasme Hospital Brussels Belgium

**Keywords:** Type-III Bare Lymphocyte Syndrome, MHC class-II deficiency, MHC class II, T-cell selection, T-cell repertoire, TcR V-gene segment use

## Abstract

Previously, we and others have shown that MHC class-II deficient humans have greatly reduced numbers of CD4+CD8– peripheral T cells. These type-III Bare Lymphocyte Syndrome patients lack MHC class-II and have an impaired MHC class-I antigen expression. In this study, we analyzed the impact of the MHC class-II deficient environment on the TCR V-gene segment usage in this reduced CD4+CD8– T-cell subset. For these studies, we employed TcR V-region-specific monoclonal antibodies (mAbs) and a semiquantitative PCR technique with V *α* and V *ß* amplimers, specific for each of the most known V *α*- and V *ß;*-gene region families. The results of our studies demonstrate that some of the V *α*-gene segments are used less frequent in the CD4+CD8– T-cell subset of the patient, whereas the majority of the TCR V *α*- and V *ß*-gene segments investigated were used with similar frequencies in both subsets in the type-III Bare Lymphocyte Syndrome patient compared to healthy control family members. Interestingly, the frequency of TcR V *α*12 transcripts was greatly diminished in the patient, both in the CD4+CD8– as well as in the CD4–CD8+ compartment, whereas this gene segment could easily be detected in the healthy family controls. On the basis of the results obtained in this study, it is concluded that within the reduced CD4+CD8– T-cell subset of this patient, most of the TCR V-gene segments tested for are employed. However, a skewing in the usage frequency of some of the V *α*-gene segments toward the CD4–CD8+ T-cell subset was noticeable in the MHC class-II deficient patient that differed from those observed in the healthy family controls.


Developmental Immunology, 1992, Vol. 2, pp. 227–236
Reprints available directly from the publisher
Photocopying permitted by license only

© 1992 Harwood Academic Publishers GmbH
Printed in the United Kingdom

### TCR Vα- and Vβ-Gene Segment Use in T-Cell Subcultures Derived from a Type-III Bare Lymphocyte Syndrome Patient Deficient in MHC Class-II Expression

M. LAMBERT,† M. VAN EGGERMOND,† F. MASCART,‡ E. DUPONT,§ and P. VAN DEN ELSEN†*

*†Department of Immunohematology and Blood Bank, University Hospital, 2300 RC Leiden, The Netherlands*
*‡Department of Immunohematology, Hospital St. Pierre, Brussels, Belgium*
*§Department of Immunohematology, Erasme Hospital, Brussels, Belgium*

Previously, we and others have shown that MHC class-II deficient humans have greatly reduced numbers of CD4+CD8– peripheral T cells. These type-III Bare Lymphocyte Syndrome patients lack MHC class-II and have an impaired MHC class-I antigen expression. In this study, we analyzed the impact of the MHC class-II deficient environment on the TCR V-gene segment usage in this reduced CD4+CD8– T-cell subset. For these studies, we employed TcR V-region-specific monoclonal antibodies (mAbs) and a semiquantitative PCR technique with Vα and Vβ amplimers, specific for each of the most known Vα- and Vβ-gene region families. The results of our studies demonstrate that some of the Vα-gene segments are used less frequent in the CD4+CD8– T-cell subset of the patient, whereas the majority of the TCR Vα- and Vβ-gene segments investigated were used with similar frequencies in both subsets in the type-III Bare Lymphocyte Syndrome patient compared to healthy control family members. Interestingly, the frequency of TcR Vα12 transcripts was greatly diminished in the patient, both in the CD4+CD8– as well as in the CD4–CD8+ compartment, whereas this gene segment could easily be detected in the healthy family controls. On the basis of the results obtained in this study, it is concluded that within the reduced CD4+CD8– T-cell subset of this patient, most of the TCR V-gene segments tested for are employed. However, a skewing in the usage frequency of some of the Vα-gene segments toward the CD4–CD8+ T-cell subset was noticeable in the MHC class-II deficient patient that differed from those observed in the healthy family controls.

KEYWORDS: Type-III Bare Lymphocyte Syndrome, MHC class-II deficiency, MHC class II, T-cell selection, T-cell repertoire, TcR V-gene segment use.

### INTRODUCTION

During T-cell development, positive and negative selection events occur within the thymic microenvironment that influence in part the composition of the mature peripheral T-cell compartment (Fink and Bevan, 1978; Zinkernagel et al., 1978; Sprent et al., 1988; Schwartz, 1989; Nikolic-Zugic and Bevan, 1990; Teh et al., 1991). Interactions between T-cell receptor (TcR) and MHC class-I or class-II antigens play a pivotal role in this process. As a result, only T cells bearing TcRs with moderate affinity for "self" MHC class I or class II can be found in the periphery (Marrack et al., 1988).

The avidity of a given TcR, expressed on the double-positive thymocyte (CD4+CD8+), for a MHC class-I or class-II antigen dictates the resulting single-positive phenotype (CD4+CD8– or CD4–CD8+) of the mature T cell (Kaye et al., 1989; von Boehmer et al., 1989; Teh et al., 1990). However, the specificity of TcRs for MHC class-I or -II antigens can be influenced in part by the coexpressed accessory molecules (CD4 or CD8, respectively; Robey et al., 1991). Interference in the interactions involved in this selection process has a dramatic effect on the composition of the peripheral T-cell compartment. (1) Disturbance of the TcR/MHC class-II antigen interaction in neonates due to masking of I-A antigens with anticlass-II monoclonal antibodies results in diminished numbers of peripheral CD4+CD8– T cells (Kruisbeek et al., 1983, 1985). (2) Mice that

*Corresponding author.

lack class-I heavy-chain expression due to a disrupted $\beta$2-microglobulin ($\beta$2m) gene have greatly reduced numbers of CD4–CD8+ peripheral T cells (Koller et al., 1990; Zijlstra et al., 1990).

The diversity of TcRs is generated in part through the use of various different V$\alpha$- and V$\beta$-gene segments. This V-gene segment use however is not random, but is influenced by the MHC haplotype, as has been shown in mice (Benoist and Mathis, 1989; Bill and Palmer, 1989). As a result, use of certain TcR V$\beta$-gene segment families can be strongly biased to either the CD4+CD8– or CD4–CD8+ population or completely deleted in both subsets (Liao et al., 1990; Singer et al., 1990). Analysis of the TcR V-gene segment use of clones sharing reactivity to a certain peptide showed that in mice these clones often use a limited V$\alpha$ and V$\beta$ repertoire (Fink et al., 1986; Engel and Hendrik, 1988). In man, also a restricted TcR V$\alpha$- and V$\beta$-gene segment use in clones reactive to a defined peptide has been reported (Wucherpfennig et al., 1990; Ben-Nun et al., 1991).

The impact of MHC class-II antigen expression on the selection of the peripheral T-cell repertoire in man can be studied in patients with the Bare Lymphocyte Syndrome (BLS) (also referred to as MHC class-II deficiency syndrome). This syndrome is a lethal combined immunodeficiency often associated with opportunistic infections and malabsorption. The main characteristic of this syndrome is defective MHC class-II antigen expression (type-II BLS), sometimes in combination with a reduced MHC class-I expression (type-III BLS), on cells that normally express these antigens, including those involved in thymic differentiation (Touraine et al., 1978; Schuurman et al., 1979; Schuurman et al., 1985; Rijkers et al., 1987). The lack of MHC class-II in conjunction with aberrant class-I expression was also noticeable in established EBV transformed B-cell lines and T-cell lines (Bull et al., 1990; Lambert et al., 1990; Lambert et al., 1991). Previously, we and others (Zegers et al., 1984; Rijkers et al., 1987; Lambert et al., 1991) have shown that in a human environment devoid of MHC class-II antigen expression, the numbers of peripheral CD4+CD8– T cells are greatly reduced. In addition, these CD4+CD8– cells have an *in vitro* proliferative disadvantage compared to CD4+CD8– cells derived from healthy controls (Lambert et al., 1991).

In this study, we have analyzed the impact of the MHC class-II deficient environment on the relative frequencies of TCR V$\alpha$- and V$\beta$-gene segment use in the CD4+CD8– peripheral T-cell subset of a type-III BLS patient.

### MATERIALS AND METHODS

### T-Cell Lines and Clones

T-cell lines were derived from blood samples obtained from a type-III BLS patient (MBI; A28, A2, B13, B39, CW6) and relatives (Father ZBI; A2, A11, B39, B52, CW–, DR2, DQw1, Mother KBI; A28, A26, B13, B44, Cw6, DR7, DR4, DQw2, DQw3 and a fetal sibling FBI; A26, A11, B44, B52, Cw–, DR4, DR2, DQw2, DQw1). Blood from the fetus was obtained via direct umbilical-cord puncture. Peripheral blood mononuclear cells (PBMC) were isolated by Fycoll/Isopaque gradient centrifugation and stimulated by polyclonal activation with 0.5% PHA (Wellcome) and 100 u. rIL-2 (Cetus) in the presence of irradiated (2500 rad) allogeneic feeder cells in Iscove's Modified Dulbecco's Medium (IMDM) containing 10% human AB serum. One week following the initial stimulation, small aliquots were frozen in medium containing 20% human serum and 10% DMSO for cryopreservation in liquid nitrogen.

Aliquots were thawed and stained with anti-CD4 and anti-CD8 monoclonal antibodies. CD4+CD8– and CD4–CD8+ T cells were isolated by FACS sorting. CD4+CD8– clones were established by limiting dilution of stained and sorted CD4+CD8– PBMC from the patient. Isolated cell populations were restimulated with 0.5% PHA and 100 u. rIL-2 in the presence of allogeneic feeder cells for another 2 weeks. Viable cells were isolated by Fycoll/Isopaque gradient centrifugation and CD4 and CD8 expression was determined by staining with anti-CD4 and anti-CD8 monoclonal antibodies on a FACScan.

### cDNA Synthesis and PCR

Total RNA was isolated by the RNAzol method (Cinna/Biotecx) according to the manufacturer's instructions. Five micrograms of total RNA was used for the synthesis of first-strand cDNA using oligo-dT as a primer (Promega, Madison,

Wisconsin). An aliquot of the cDNA preparation was subjected to amplification with 2.5 units of Taq polymerase (Amplitaq, Cetus Corporation, Emeryville, California) in the following reaction mixture: 20 pmol of specific V$\alpha$ or V$\beta$ primer, and 20 pmol of C$\alpha$ or C$\beta$ primer, respectively, 50 mM KCl, 10 mM Tris-HCl (pH 8.4), 4 mM MgCl2, 0.5 mM each of dNTP, and 0.06 mg/ml bovine serum albumin (BSA) in a final volume of 100 $\mu$l. Sequences of used primers were published by Wucherpfennig et al. (1990) (V$\beta$ 1 to V$\beta$ 16 except V$\beta$12) and by Oksenberg et al. (1990) (V$\alpha$2, 3, 5–9, 11, 12). The V$\alpha$17–21 oligonucleotides have been described as V$\alpha$13–17 by Oksenberg et al. (1990). Sequences of additional primers were

| *Primer* | *Sequence* |
|---|---|
| V$\beta$12 | 5'CTGAGATGTCACCAGACTGAGAACCACCGC 3' |
| V$\beta$17 | 5'GCACAAGAAGCGATTCTCATCTCAATGCCC 3' |
| V$\beta$18 | 5'CATCTGTCTTCTGGGGGCAGGTCTCTCAAA 3' |
| V$\beta$19 | 5'ATAGCTGAAGGGTACAGCGTCTCTCGGGAG 3' |
| V$\beta$20 | 5'TCTAATATTCATCAATGGCCAGCGACCCT 3' |
| 5'C$\beta$ | 5'CCGAGGTCGCTGTGTTTGAGCCAT 3' |
| 3'C$\beta$ | 5'CTCTTGACCATGGCCATC 3' |
| V$\alpha$1 | 5'TTGCCCTGAGAGATGCCAGAG 3' |
| V$\alpha$4 | 5'AAGACAGAAAGTCCAGTACCTTGATCCTGC 3' |
| V$\alpha$13 | 5'TGCTGTGTGAGAGGAATACAAGTG 3' |
| V$\alpha$14 | 5'GATCTCCACCTGTCTTGAATTTAG 3' |
| V$\alpha$15 | 5'CAGAGTCTTTTCCTGAGTGTCCGAG 3' |
| V$\alpha$16 | 5'GAGTGGGCTGAGAGCTCAGTCAGTG 3' |
| V$\alpha$22 | 5'ATGTCAGGCAATGACAAGGGAAGC 3' |
| 5'C$\alpha$ | 5'GAACCCTGACCCTGCCGTGTACC 3' |
| 3'C$\alpha$ | 5'ATCATAAATTCGGGTAGGATCC 3' |

The profile of the PCR cycles was as follows: denaturation at 95 °C for 60 s, annealing at 55 °C for 60 s and elongation at 72 °C for 60 s in a Bio-Med Thermocycler 60. Amplifications were performed for a maximum of 40 cycles to determine trace amounts of specific V-gene segment usage. After 25 (V$\alpha$) or 20 (V$\beta$) cycles of amplification, aliquots were drawn and used for Southern blot analysis. The PCR products were size fractionated on a 0.8% agarose gel and transferred to Gene Screen Plus (NEN). TcR-specific DNA sequences were detected by hybridization using an internal $^{32}$P labeled C$\alpha$ or C$\beta$ probe, respectively (Yoshikai et al., 1984; Yanagi et al., 1985). Transfer and hybridization were performed according to the manufacturer's instructions.

MHC class II transcription analysis (DR, DP, and DQ A and B chains) was performed as described (Lambert et al., 1991). Transfer and hybridizations were performed as described before. MHC class-II specific sequences were visualized using probes from the Xth International HLA workshop.

### Flow Microfluorometric Analysis

Cells were analyzed by indirect immunofluorescence on a flow cytometer (FACScan, Becton-Dickinson). Cells were washed with PBS containing 1% BSA and 0.1% sodium azide. Aliquots of 1 $\times 10^5$ cells were incubated at 4 °C with monoclonal antibodies (mAb) for 30 min, either directly analyzed when conjugated mAbs were used, or washed and reincubated with fluorescein-isothiocyanate-conjugated rabbit antimouse immunoglobulin F(ab)$^2$ fragments (Dakopatts). After another 30 min, cells were washed three times and analyzed. The used monoclonal antibodies were anti-CD4$^{Fitc}$ and anti-CD8$^{PE}$ (Becton-Dickinson) and antibodies that are specific for family members belonging to the TcR V$\beta$5-, V$\beta$6-, V$\beta$8-, V$\beta$12-, and V$\alpha$2-gene segment family (Diversi-T, T cell Science) and V$\alpha$12 (DerSimonian et al., 1991).

### RESULTS

### Immunofluorescence Analysis

In previous studies, we have shown that despite the apparent lack of MHC class-II antigen expression in the type-III Bare Lymphocyte Syndrome (BLS) patient MBI, CD4+CD8− T cells can be found among PBMC, albeit their numbers are reduced (ratio CD4+/CD8+=0.75; Lambert et al., 1991). Of interest was the observation that within the CD4+CD8− subset, 48% of the CD4+CD8− T cells coexpressed the CD45RO marker, which is characteristic for activated T cells. As shown in Fig. 1, the T-cell lines derived from patient MBI (type-III BLS) lacked detectable levels of MHC class-II transcripts as determined by polymerase chain reaction (PCR) as well as staining with anti-HLA class-II specific monoclonal antibodies (Lambert et al., 1991).

To address the question whether the lack of MHC class-II antigen expression has an impact on the composition of this impaired peripheral CD4+ T-cell subset with respect to employment of the various TcR V-gene segments, we studied the frequency of TcR V$\alpha$- and V$\beta$-gene segment use in these cells. PBMC were stained with mon-

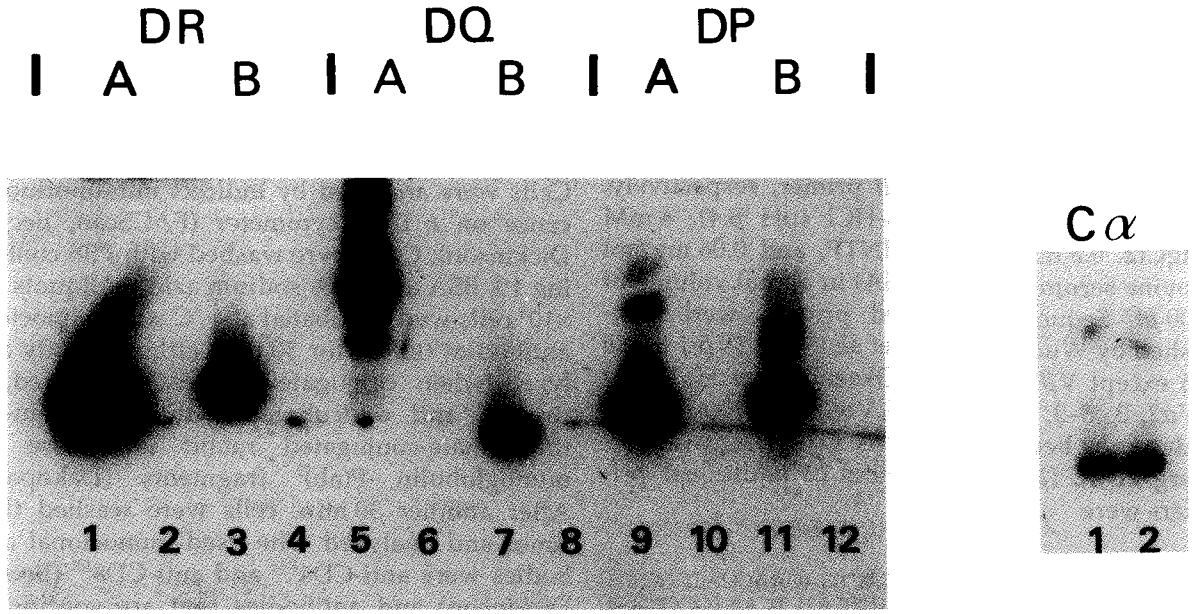

FIGURE 1. MHC class-II expression in T-cell lines derived from patient MBI (lanes 2, 4, 6, 8, 10, 12) and healthy control KBI (lanes 1, 3, 5, 7, 9, 11) by PCR/Southern analysis. 1-2: DR-A; 3-4: DR-B; 5-6: DQ-A; 7-8: DQ-B; 9-10: DR-A; 11-12: DP-B; and Cα (1: KBI; 2: BI).

TABLE 1
Flow Cytometric Analysis of TcR Expression on PBMC Derived from MHC Class-II Deficient Patient (MBI) and Two Healthy Family Controls (ZBI, KBI)[a]

| | Vα2 | Vβ5a | Vβ5b | Vβ6 | Vβ8 | Vβ12 |
|---|---|---|---|---|---|---|
| MBI | 3.7 | 7.4 | 5.1 | 5.6 | 11.0 | 1.8 |
| KBI | 2.8 | 2.8 | 3.8 | 2.0 | 7.0 | 2.5 |
| ZBI | 4.7 | 7.0 | 2.5 | 5.2 | 8.4 | 4.3 |

[a]Results are expressed as percentage positive cells.

oclonal antibodies specific for the TcR Vβ5-, 6-, 8-, 12- and Vα2-gene segment families. The results of these analyses, summarized in Table 1, show that all tested TcR V-region families were expressed in the patient-derived PBMC. The observed frequencies in the patient-derived material were within normal range. Variation in the frequency of use of the different V-gene families was noticeable when the different individuals were compared. In general, the percentage of T cells carrying a particular Vβ-gene segment was always less than 10%, except for Vβ 8 in the patient-derived PBMC (see Table 1). The relative high frequency of Vβ8 positive cells seen in the patient and healthy controls was also observed in PBMC derived from other, unrelated individuals using the same antibody (data not shown).

To determine the TcR Vβ and Vα use in the individual CD4+CD8– and CD4–CD8+ T-cell populations, *in vitro* expanded T cells were isolated by FACS sorting and analyzed (Table 2). In the patient-derived material, all tested Vα- and

TABLE 2
Flow Cytometric Analysis of TcR Expression on *In Vitro* Activated CD4+CD8– and CD4–CD8+ T Cells Derived from a Bare Lymphocyte Type-III patient (MBI) and Two Healthy Family Controls (ZBI, KBI)[a]

| | | Vα2 | Vα12 | Vβ5a | Vβ5b | Vβ6 | Vβ8 | Vβ12 |
|---|---|---|---|---|---|---|---|---|
| MBI | CD4+ | 3.5 | 0.5 | 2.5 | 2.0 | 3.4 | 4.1 | 2.6 |
| | CD8+ | 7.0 | 0.4 | 1.9 | 0.9 | 3.4 | 5.7 | 1.6 |
| KBI | CD4+ | 2.4 | n.t. | 1.4 | 1.0 | 2.0 | 6.7 | 1.2 |
| | CD8+ | 0.1 | 2.7 | 9.9 | 6.5 | 0.2 | 0.8 | 0.1 |
| ZBI | CD4+ | 4.3 | 2.3 | 3.6 | 1.7 | 5.2 | 13.0 | 4.1 |
| | CD8+ | 4.4 | 5.2 | 2.3 | 1.5 | 3.7 | 8.9 | 1.8 |

[a]Results are expressed as percentage positive cells.

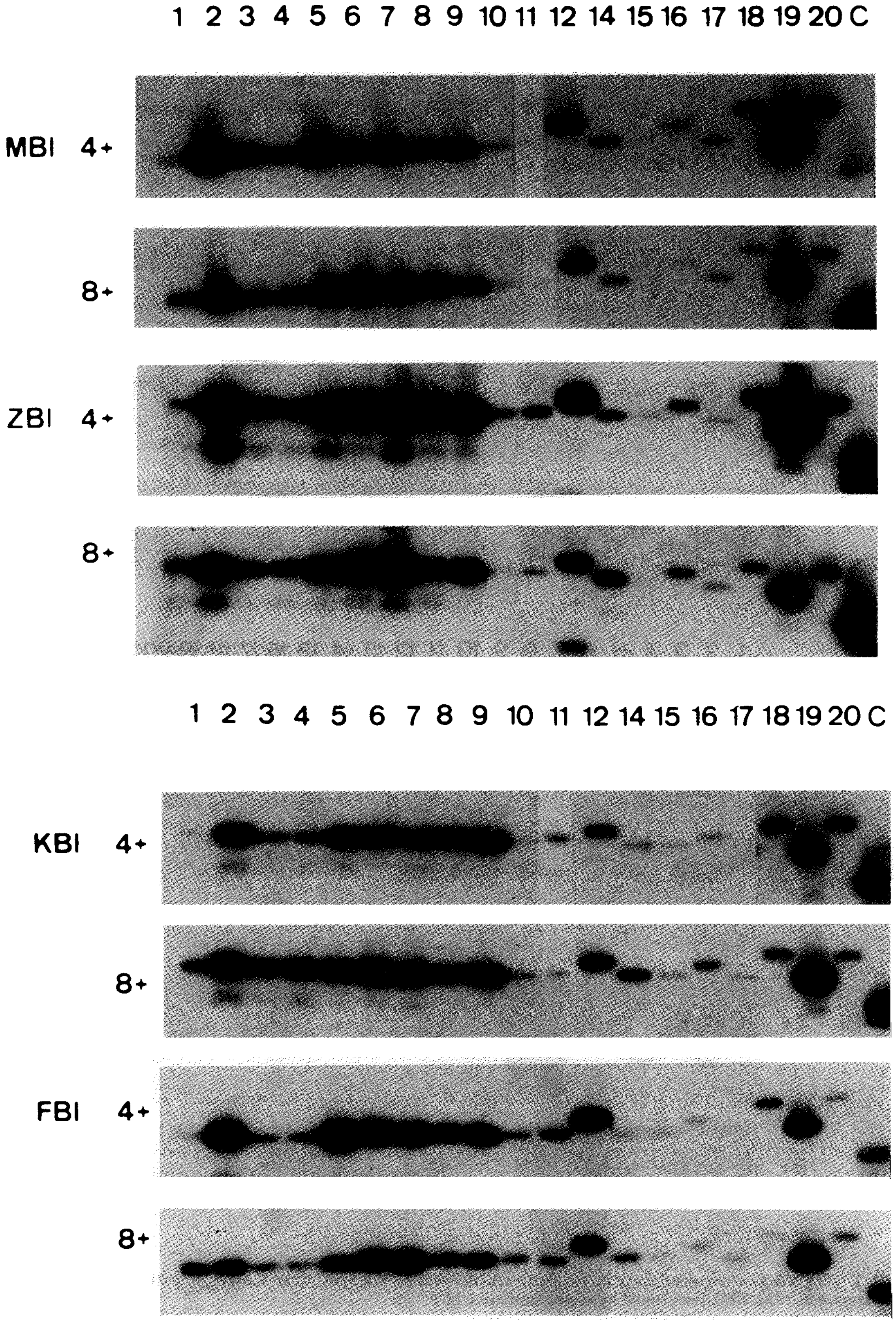

FIGURE 2. TcR Vβ-gene segment usage in T-cell cultures derived from a MHC class-II deficient patient (MBI), a healthy sibling (FBI), and parents (KBI, ZBI) determined by semiquantitative PCR.

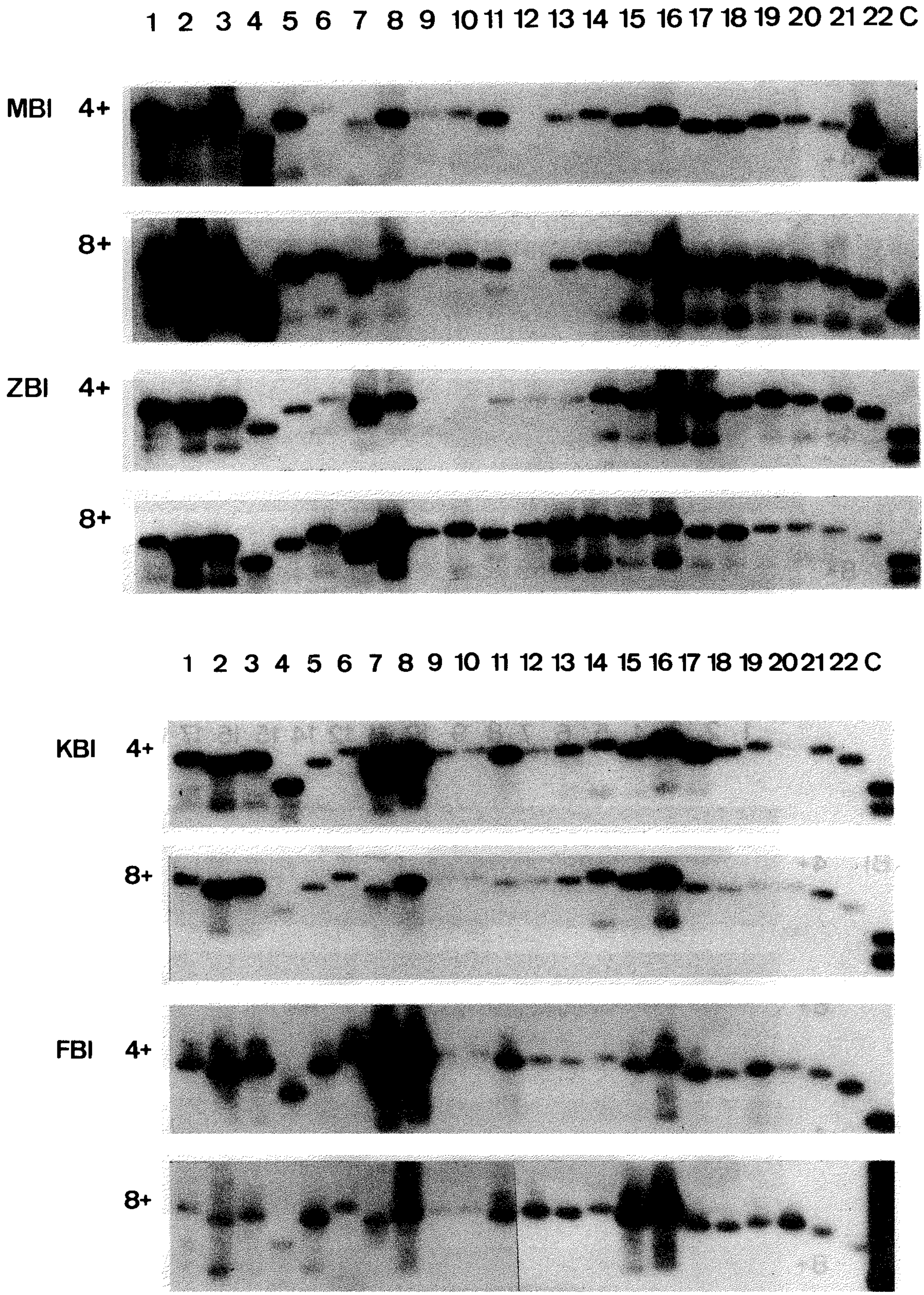

FIGURE 3. TcR V$\alpha$-gene segment usage in T-cell cultures derived from a MHC class-II deficient patient (MBI), a healthy sibling (FBI), and parents (KBI, ZBI) determined by semiquantitative PCR.

Vβ-gene segment families could be detected both in the CD4 and CD8 positive population with the exception of Vα12. The frequency of T cells reactive with the monoclonal antibodies used was in most cases comparable with the frequencies found in the cell cultures derived from the non-MHC class-II deficient relatives. Of note is the apparent skewing of the Vα2 and Vβ8 V-regions toward the CD4–CD8+ subset in the MHC class-II deficient patient that was not observed in material derived from both healthy family members (Table 2).

### TcR V-Gene Segment Usage as Determined by PCR Analysis

*Vβ-gene family usage*

Next, we have expanded our analyses of the TCR V-gene segment use at the transcriptional level by PCR. To this end, 19 different oligonucleotides were used to amplify the different Vβ-gene segment families. The results of these PCR analyses show that both in the CD4+CD8– and CD4–CD8+ T-cell subsets, nearly all TCR Vβ families tested could be detected (Fig. 2). In general, TcR Vβ-gene segment families with the most members showed the strongest amplification. An exception was Vβ19, which has only one member and was strongly amplified in all individuals, both in the CD4+CD8– and CD4–CD8+ T-cell population. Some TcR Vβ-gene segment families were used at almost equal frequencies in both CD4+ and CD8+ populations in all individuals, whereas other Vβ-gene segment families showed an apparent skewing to one subpopulation in some but not all individuals. For instance, Vβ1 showed a skewing to the CD4–CD8+ population in KBI, FBI, and MBI, whereas Vβ14 was preferentially used in the CD4–CD8+ population in KBI, FBI, and ZBI but not MBI. Likewise, Vβ18 was skewed to the CD4+CD8– population in FBI, MBI, and ZBI (Fig. 2).

*TcR Vα-gene family usage.*

To study the TcR Vα-gene segment use, 22 different Vα-gene segment families were analyzed. The results of these studies are depicted in Fig. 3. Differences in the frequency of use of the various TcR Vα families are more pronounced in the CD4+CD8– and CD4–CD8+ populations within one individual, compared to the TcR Vβ-gene segment use (Fig. 3). For instance, in the sibling FBI, Vα15 and Vα16 seem to be more frequently used in the CD4–CD8+ subset, whereas in the father ZBI, amplification of Vα15 and 16 was comparable in both subsets (Fig. 3). In the CD4+CD8– subculture of the type-III BLS patient, a clear diminished use of the Vα1, 2, 3, 7 families could be observed, which was not seen in the healthy family controls in addition to biased expression in the CD4–CD8+ subset of various other TcR Vα-gene segments not seen in healthy controls. The most striking difference, however, was the apparent low frequency of use of the TcR Vα12-gene segment in the patient both in the CD4+CD8– and CD4–CD8+ subsets.

TABLE 3
Analysis of TcR Vα and Vβ Usage in CD4+CD8– T-Cell Clones Derived from a MHC Class-II Deficient Patient[a]

| Clone | Vα | Vβ |
|---|---|---|
| M4C2 | N.D. | 19 |
| M4C3 | 2, 4 | 10 |
| M4C4 | 4 | 9 |
| M4C5 | 11 | 3 |
| M4C8 | 4 | 3 |
| M4C9 | 15 | 20 |
| M4C10 | 17 | 19 |
| M4C11 | 8 | 2 |
| M4C12 | 21, 2 | 19 |
| M4C13 | 3 | 7 |
| M4C16 | 10 | 15 |
| M4C17 | 11 | 7 |
| M4C18 | 4 | 5 |
| M4C19 | 4 | 5 |

TcR Vα- and Vβ-gene segment usage was determined by PCR analysis.

To confirm the random TcR Vα and Vβ use in the CD4+CD8– T-cell subset in the type-III BLS patient, the V-gene segment use in TcR expressed on several CD4+CD8– T-cell clones was analyzed. The clonality of these clones was tested by gene rearrangement analysis of the TcRβ chain using the restriction enzymes Eco RI and Hind III and a Cβ probe (data not shown). As in the bulk culture, no preferential or restricted use of a given TcR Vα- and Vβ-gene family could be detected in these clones (Table 3). Of note is that in three clones, the Vβ19-gene segment could be amplified specifically, which might account for the preferential use observed in the analysis of the bulk culture. As in the CD4+CD8– and CD4–CD8+ bulk cultures, none of the clones analyzed used the TcR Vα12-gene segment.

### DISCUSSION

The type-III Bare Lymphocyte Syndrome provides a human model to study the influence of the lack of MHC class-II antigen expression on the composition of the peripheral T-cell compartment. In this study, we have analyzed the impact of the MHC class-II deficient environment on the TcR V-gene segment usage in the CD4+CD8– and CD4–CD8+ T-cell subsets. Our analysis showed that for most of the V regions tested, no restricted or unusual skewing in the frequency of employment of the various TcR V$\alpha$- and V$\beta$-gene segments could be detected. Both within the CD4+CD8– as well as in the CD4–CD8+ T-cell subset of the patient, the observed frequency of TCR V-gene use for the majority of the TCR V-gene segments tested was comparable to the healthy family controls. However, in the patient, most of the V$\alpha$-gene segments were used at higher frequencies in the CD4–CD8+ T-cell subset, in particular, the V$\alpha$1, 2, 3, and 7 genes. Of note is that the V$\alpha$12-gene segment was used at much lower frequencies both in the CD4+CD8– and CD4–CD8+ subsets, in comparison to healthy controls.

Our studies showed that in the frequency of TcR V$\alpha$-gene segment use, more variation was noticeable compared to V$\beta$-gene segment use between the CD4+CD8– T cells and CD4–CD8+ T cells within one individual. This could be related because most of the V$\alpha$-gene segment families contain only one member, whereas the majority of the V$\beta$-gene segment families have more members. As a result, differences in usage frequency of the different V$\beta$ subfamilies can be masked by the amplification of other members of the same family. The results from this study indicate that in man, the impact of MHC class-II antigen expression on T-cell development is more pronounced on the TcR V$\alpha$- rather than on the V$\beta$-gene segment use. This in spite of the fact that both the TcR V$\alpha$ and V$\beta$ chain seem to be involved in binding of the peptide/MHC complex (Chotia et al., 1988; Davis and Bjorkman, 1988).

Although our analyses show variety in TcR V$\alpha$- and V$\beta$-gene segment use between the single CD4+CD8– and CD4–CD8+ T-cell population, the skewing of a particular V-gene segment to a subset is less strong as observed in some mouse strains. Several factors could contribute to this difference between man and mouse. First, the composition of the peripheral T-cell population is not only influenced by the individual haplotype, but also by the exposure to environmental antigens and immunological activation at a given moment. It should be noted that the analyzed mice of various strains have been kept in a germ-free state. Second, exposure to "self-super-antigens" results in a skewing of certain TcR V$\beta$-gene segment positive T-cells to either the CD4–CD8+ or CD4+CD8– subset (Benoist and Mathis, 1989; Bill and Palmer, 1989; Blackman et al., 1989; Liao et al., 1989) or to a complete deletion of T cells bearing a TcR with certain V$\beta$-gene segments in both the CD4+CD8– and CD4–CD8+ population (Kappler et al., 1988; Kisielow et al., 1988; MacDonald et al., 1988a, 1988b; Pircher et al., 1989). In mice several "self super-antigens" are identified as endogenous retroviral sequences (Dyson et al., 1991; Frankel et al., 1991; Marrack et al., 1991; Woodland et al., 1991), but no human analogue has been found so far. Third, in mice, lack of use of a particular V-gene segment also resulted from deletion of part of the V-gene segments on the genomic level as determined by Southern blot analysis of the TcR loci both in inbred and in wild mice (Behlke et al., 1986; Haqqi et al., 1989a, 1989b; Pullen et al., 1990). In man, no major deletion of V-gene segments could be detected in most individuals (Concannon et al., 1987; Baccala et al., 1991). Fourth, most of the analyzed mice are homozygous, whereas our analyzed individuals are heterozygous. This heterozygocity could mask the influence of the haplotype on the peripheral T-cell compartment.

The presence of single CD4+CD8– T cells in the periphery of this MHC class-II deficient patient is in seemingly contradiction with studies in the mouse in which interference in the TcR/MHC class-II interaction leads to an almost complete lack of CD4+CD8– T cells in the periphery (Kruisbeek et al., 1983, 1985). Recently, MHC class-II deficient mice have been generated by homologous recombination. Here, the lack of MHC class-II antigen expression in these mice resulted in a strong reduction in the number of single CD4+CD8– T cells in the periphery (Cosgrove et al., 1991; Grusby et al., 1991). Of note is the observation that despite the lack of expression of the to-date known mouse MHC class-II structures that are responsible for the generation of the CD4+CD8− T-cell subset, the periphery of these mice is not devoid of CD4+CD8− T cells. In these mice, small numbers of CD4+CD8− T cells can be found that exhibit random use of the TCR V-gene segments tested for (Cosgrove et al., 1991; Grusby et al., 1991). These observations are reminiscent of the results from this study in which we have demonstrated that in a human model with no detectable peripheral MHC class-II expression, CD4+CD8− T cells can be found in the periphery, albeit their numbers are greatly reduced. Furthermore, these CD4+CD8− T cells are not restricted in their TcR V-gene segment use, indicating that these cells are not derived from an oligoclonal outgrowth of a few CD4+CD8− T cells that have escaped from the thymic selection process. It is at present not clear whether the observed skewing in the use frequency of some of the TcR V-gene segments in the T-cell subsets is a direct consequence of aberrant thymic selection processes or that it is resulting from a lack of MHC class-II mediated postthymic modification of the peripheral T-cell compartment.

### ACKNOWLEDGMENTS

This research was supported in part by the Macropa Foundation and the J.A. Cohen Institute for Radiopathology and Radiation Protection (IRS). The authors thank Drs. J. Kurnick, F. Konig, and C. Melief for critically reading the manuscript, Drs. H. DerSimonian and M. Brenner for making the Vα12-specific monoclonal antibody available and to Dr. J. J. van Rood for his generous support.

*(Received September 19, 1991)*

*(Accepted November 18, 1991)*

### REFERENCES

Baccala R., Kono D.H., Walker S., Balderas R.S., and Theofilopoulos A.N. (1991). Genomically imposed and somatically modified human thymocyte Vβ gene repertoires. Proc. Natl. Acad. Sci. USA **88:** 2908–2912.

Behlke M., Chou H., Huppi K., and Loh D. (1986). Murine T cell receptor mutants with deletions of β-chain variable region genes. Proc. Natl. Acad. Sci. USA **83:** 767–771.

Ben-Nun A., Liblau R.S., Cohen L., Lehmann D., Tournier-Lasserve E., Rosenzweig A., Jingwu Z., Raus J.C.M., and Bach M.A. (1991). Restricted T-cell receptor Vβ gene usage by myelin basic protein-specific T-cell clones in multiple sclerosis: Predominant genes vary in individuals. Proc. Natl. Acad. Sci. USA **88:** 2466–2470.

Benoist C., and Mathis D. (1989). Positive selection of the T cell repertoire: Where and when does it occur? Cell **58:** 1027–1033.

Bill J., and Palmer E. (1989). Positive selection of CD4+ T cells mediated by MHC class II-bearing stromal cells in the thymic cortex. Nature **341:** 649–651.

Blackman M.A., Marrack P., and Kappler J. (1989). Influence of the major histocompatibility complex on positive thymic selection of Vβ17a+ T cells. Science **244:** 214–217.

Bull M., Van Hoef A., and Gorski J. (1990). Transcription analysis of class II human leukocyte antigen genes from normal and immunodeficient B lymphocytes, using polymerase chain reaction. Mol. Cell. Biol. **10:** 3792–3796.

Chothia C., Boswell D.R., and Lesk A.M. (1988). The outline structure of the T-cell αβ receptor. EMBO J. **7:** 3745–3755.

Concannon P., Gatti R.A., and Hood L.E. (1987). Human T-cell receptor Vβ gene polymorphism. J. Exp. Med. **165:** 1130–1140.

Cosgrove D., Gray D., Dierich A., Kaufman J., Lemeur M., Benoist C., and Mathis D. (1991). Mice lacking MHC class II molecules. Cell **66:** 1051–1066.

Davis M.M. and Bjorkman P.J. (1988). T-cell antigen receptor genes and T-cell recognition. Nature **334:** 395–402.

DerSimonian H., Band H., and Brenner M.B. (1991). Increased frequency of T-cell receptor Vα12.1 expression on CD8+ T-cells: Evidence that Vα participates in shaping the peripheral T-cell repertoire. J. Exp. Med. **174:** 639–648.

Dyson P.J., Knight A.M., Fairchild S., Simpson E., and Tomonari K. (1991). Genes encoding ligands for deletions of Vβ11 T-cells cosegregate with mammary tumour virus genomes. Nature **349:** 531–532.

Engel I., and Hedrick S.M. (1988). Site-directed mutations in the VDJ junctional region of a T-cell receptor β chain cause changes in antigenic peptide recognition. Cell **54:** 473–484.

Fink P.J. and Bevan M.J. (1978). H-2 antigens of the thymus determine lymphocyte specificity. J. Exp. Med. **148:** 766–775.

Fink P.J., Matis L.A., McElligott D.L., Bookman M.A., and Hedrick S.M. (1986). Correlations between T-cell specificity and the structure of the antigen receptor. Nature **321:** 219–226.

Frankel W.N., Rudy C., Coffin J.M., and Hubert B.T. (1991). Linkage of Mls genes to endogenous mammary tumour viruses of inbred mice. Nature **349:** 526–528.

Grusby M.J., Johnson R.S., Papaioannou V.E., and Glimcher L.H. (1991). Depletion of CD4+ T cells in major histocompatibility complex class II-deficient mice. Science **253:** 1417–1420.

Haqqi T.M., Banerjee S., Anderson G., and David C.S. (1989a). RIII S/J (H-2r). An inbred mouse strain with a massive deletion of T cell receptor Vβ genes. J. Exp. Med. **169:** 1903–1909.

Haqqi T.M., Banerjee S., Jones W.L., Anderson G., Behlke M.A., Loh D.Y., Luthra H.S., and David C.S. (1989b). Identification of T cell receptor Vβ deletion mutant mouse strain AU/ssJ (H-2q) which is resistant to collagen-induced arthritis. Immunogenetics **29:** 180–185.

Kappler J., Staerz U., White H., and Marrack P. (1988). Self-tolerance eliminates T cells specific for Mls-modified products of the major histocompatibility complex. Nature **332:** 35–40.

Kaye J., Hsu M.L., Sauron M.E., Jameson S.C., Gascoigne N.R.J., and Hedrick S.M. (1989). Selective development of CD4+ T cells in transgenic mice expressing a class II MHC-restricted antigen receptor. Nature **341:** 746–749.

Kisielow P., Bluthmann J., Staerz U.D., Steinmetz M., and von Boehmer H. (1988). Tolerance in T-cell receptor transgenic mice involves deletion of nonmature CD4+CD8+ thymocytes. Nature **333:** 742–746.

Koller B.H., Marrack P., Kappler J.W., and Smithies O. (1990). Normal development of mice deficient in $\beta$2m MHC class I proteins, and CD8+ T cells. Science **248:** 1227–1230.

Kruisbeek A.M., Fultz M.J., Sharrow S.O., Singer A., and Mond J.J. (1983). Early development of the T cell repertoire. *In vivo* treatment of neonatal mice with anti-I-A antibodies interferes with differentiation of I-restricted T cells but not K/D-restricted T cells. J. Exp. Med. **157:** 1932–1946.

Kruisbeek A.M., Mond J.J., Foulkes B.J., Carmen J.A., Bridges S., and Long D.L. (1985). Absence of the Lyt-2-, L3T4+ lineage of T cells in mice treated neonatally with anti-I-A correlates with the absence of intrathymic I-A bearing antigen presenting cell function. J. Exp. Med. **161:** 1029–1047.

Lambert M., Van Eggermond M.C.J.A., Kraakman M.E.M., Schuurman R.K.B., and Van den Elsen P.J. (1990). The MHC class II deficiency syndrome: Heterogeneity at the level of the response to 5-azadeoxycytidine. Res. Immunol. **141:** 129–140.

Lambert M., Van Eggermond M.C.J.A., Andrien M., Mascart F., Vamos E., Dupont E., and Van den Elsen P.J. (1991). Analysis of the peripheral T cell compartment in the MHC class II deficiency syndrome. Res. Immunol. **142:** 789–798.

Liao N.S., Maltzman J., and Raulet D.H. (1989). Positive selection determines T cell receptor V$\beta$14 gene usage by CD8 T cells. J. Exp. Med. **170:** 135–143.

Liao N.S., Maltzman J., and Raulet D.H. (1990). Expression of the V$\beta$5.1 gene by murine peripheral T cells is controlled by MHC genes and skewed to the CD8+ subset. J. Immunol. **144:** 844–848.

MacDonald H.R., Pedrazzini T., Schneider R., Louis J.A., Zinkernagel R.M., and Hengartner H. (1988a). Intrathymic elimination of Mls$^a$-reactive (V$\beta$6+) cells during neonatal tolerance induction to Mls$^a$ encoded antigens. J. Exp. Med. **167:** 2005–2010.

MacDonald H.R., Schneider R., Lees R.K., Howe R.C., Acha-Orbea H., Festenstein H., Zinkernagel R.M., and Hengartner H. (1988b). T-cell receptor V$\beta$ use predicts reactivity and tolerance to Mls$^a$-encoded antigens. Nature **332:** 40–45.

Marrack P., Kushnir E., and Kappler J. (1991). A maternally inherited superantigen encoded by a mammary tumour virus. Nature **349:** 524–526.

Marrack P., Lo D., Brinster R., Palmiter R., Burkly L., Flavell R.H., and Kappler J. (1988). The effect of thymus environment on T cell development and tolerance. Cell **53:** 627–634.

Nikolic-Zugic J., and Bevan M.J. (1990). Role of self peptides in positively selecting the T-cell repertoire. Nature **344:** 65–67.

Oksenberg J.R., Stuart S., Begovich A.B., Bell R.B., Erhlich H.A., Steinman L., and Bernard C.C.A. (1990). Limited heterogeneity of rearranged T-cell receptor V$\alpha$ transcripts in brains of multiple sclerosis patients. Nature **345:** 344–346.

Pircher H., Mak T.W., Long R., Balhausen W., Ruedi E., Hengartner H., Zinkernagel R.M., and Burki K. (1989). T cell tolerance to Mls$^a$ encoded antigen in T cell receptor V$\beta$8.1 chain transgenic mice. EMBO J. **8:** 719–727.

Pullen A.M., Potts W., Wakeland E.K., Kappler J., and Marrack P. (1990). Surprisingly uneven distribution of the T cell receptor V$\beta$ receptor in wild mice. J. Exp. Med. **171:** 49–62.

Rijkers G.T., Roord J.J., Koning F., Kuis W., and Zegers B.J.M. (1987). Phenotypical and functional analysis of B lymphocytes of two siblings with combined immunodeficiency and defective expression of major histocompatibility complex (MHC) class II antigens on mononuclear cells. J. Clin. Immunol. **7:** 98–106.

Robey E., Ramsdell F., Elliott J., Raulet D., Kioussis D., Axel R., and Fowlkes B.J. (1991). Expression of CD4 in transgenic mice alters the specificity of CD8 cells for allogeneic major histocompatibility complex. Proc. Natl. Acad. Sci. USA **88:** 608–612.

Schuurman H.J., Van den Wijngaert F.P., Huber J., Schuurman R.K.B., Zegers B.J.M., Roord J.J., and Kater L. (1985). The thymus in "Bare Lymphocyte" Syndrome: Significance of expression of major histocompatibility complex antigens on thymic epithelial cells in intrathymic T-cell maturation. Human Immunol. **13:** 69–82:

Schuurman R.K.B., Roord J.J., Vossen J.M., Schellekens P.T.A., Feltkamp-Vroom T.M., Doyer E., Gmelig-Meyling F., and Visser H.K.A. (1979). Failure of lymphocyte membrane HLA-A and -B expression in two siblings with combined immunodeficiency. Clin. Immunol. Immunopath. **14:** 418–434.

Schwartz R.H. (1989). Acquisition of immunologic self tolerance. Cell **57:** 1073–1081.

Singer P.A., Balderas R.S., and Theofilopoulos A.N. (1990). Thymic selection defines multiple T cell receptor V$\beta$ "repertoire phenotypes" at the CD4/CD8 subset level. EMBO J. **9:** 3641–3648.

Sprent J., Lo D., Gao E.K., and Ron Y. (1988). T cell selection in the thymus. Immunol. Rev. **101:** 173–190.

Teh H.-S., Garvin A.M., Forbush K.A., Carlow D.A., and Perlmutter R.M. (1991). Participation of CD4 coreceptor molecules in T-cell repertoire selection. *Nature* **349:** 241–243.

Teh H.S., Kishi H., Scott B., Borgulya P., von Boehmer H., and Kisielow P. (1990). Early deletion and late positive selection of T cells expressing a male-specific receptor in T-cell receptor transgenic mice. Dev. Immunol. **1:** 1–10.

Touraine J.-L., Betuel H., Suillet G., and Jeune M. (1978). Combined immunodeficiency disease associated with absence of cell surface HLA-A and -B antigens. J. Pediatr. **93:** 47–51.

Von Boehmer H., Kisielow P., Kishi H., Scott B., Borgulya P., and Teh H.S. (1989). The expression of CD4 and CD8 accessory molecules on mature T cells is not random but correlates with the specificity of the $\alpha\beta$ receptor for antigen. Immunol. Rev. **109:** 143–149.

Woodland D.L., Happ M.P., Gollob K.J., and Palmer E. (1991). An endogenous retrovirus mediating deletion of $\alpha\beta$ T cells? Nature **349:** 529–530.

Wucherpfennig K.W., Ota K., Endo N., Seidman J.G., Rosenzweig A., Weiner H.L., and Hafler D.A. (1990). Shared human T cell receptor V$\beta$ usage to immunodominant regions of myelin basic protein. Science **248:** 1016–1019.

Yanagi Y., Chan A., Chin B., Minden M., and Mak T.W. (1985). Analysis of cDNA clones specific for human T-cells and the $\alpha$ and $\beta$ chains of the T-cell receptor heterodimer from a human T-cell lines. Proc. Natl. Acad. Sci. USA **82:** 3430–3434.

Yoshikai Y., Anatoniou D., Clark S.P., Yanagi Y., Angsterm R., Van den Elsen P., Terhorst C., and Mak T.W. (1984). Sequence and expression of transcripts of the human T-cell receptor $\beta$ chain genes. Nature **321:** 521–524.

Zegers B.J.M., Heijnen C.J., Roord J.J., Kuis W., Stoop J.W., and Ballieux R.R. (1984). Combined immunodeficiency with defective expression of HLA-antigens: Analysis of the nature of the defective monocyte T-cell interaction. *In:* Progress in immunodeficiency research and therapy I, Griscelli C., and Vossen J.M., Eds. (Amsterdam: Excepta Medica Elsevier), pp. 35–42.

Zijlstra M., Bix M., Simister N.E., Loring J.M., Raulet D.H., and Jaenisch R. (1990). $\beta$2-microglobulin deficient mice lack CD4-8+ cytolytic T-cells. Nature **344:** 742–746.

Zinkernagel R.M., Callahan G.N., Althage A., Cooper S., Klein P.A., and Klein J. (1978). On the thymus in the differentiation of "H-2 self-recognition" by T-cells: Evidence for dual recognition? J. Exp. Med. **147:** 882–896.

Zuniga-Pflucker J.C., Longo D.L., and Kruisbeek A.M. (1989). Positive selection of CD4–CD8+ T cell in the thymus of normal mice. Nature **338:** 76–78.

